# Supplementary material for: Modeling glioblastoma heterogeneity as a dynamic network of cell states
Source: Mol Syst Biol. 2021 Sep 16;17(9):e10105. doi: 10.15252/msb.202010105 (PMC8444284; doi:10.15252/msb.202010105)
Supplement: Supplementary file 6 — Source Data for Figure 5 [file MSB-17-e10105-s004.zip › Figure5A_sourcedata/GSEA_3071/nosigdb_stateI.GseaPreranked.1622795145664/WANG_MESENCHYMAL_PMID_28697342.html]

Details for gene set WANG\_MESENCHYMAL\_PMID\_28697342[GSEA]

|  || Dataset | state13071 |
| Phenotype | NoPhenotypeAvailable |
| Upregulated in class | na\_pos |
| GeneSet | WANG\_MESENCHYMAL\_PMID\_28697342 |
| Enrichment Score (ES) | 0.7074676 |
| Normalized Enrichment Score (NES) | 2.9005542 |
| Nominal p-value | 0.0 |
| FDR q-value | 0.0 |
| FWER p-Value | 0.0 |
Table: GSEA Results Summary

  

Fig 1: Enrichment plot: WANG\_MESENCHYMAL\_PMID\_28697342      
 Profile of the Running ES Score & Positions of GeneSet Members on the Rank Ordered List

  

| PROBE | GENE SYMBOL | GENE\_TITLE | RANK IN GENE LIST | RANK METRIC SCORE | RUNNING ES | CORE ENRICHMENT || 1 | COL3A1 |  |  | 3 | 2.646 | 0.1183 | Yes |
| 2 | LUM |  |  | 4 | 2.203 | 0.2175 | Yes |
| 3 | COL1A1 |  |  | 6 | 2.008 | 0.3076 | Yes |
| 4 | COL5A1 |  |  | 14 | 1.706 | 0.3824 | Yes |
| 5 | TGFBI |  |  | 17 | 1.674 | 0.4572 | Yes |
| 6 | COL15A1 |  |  | 25 | 1.550 | 0.5249 | Yes |
| 7 | P4HA2 |  |  | 82 | 1.171 | 0.5616 | Yes |
| 8 | NNMT |  |  | 88 | 1.132 | 0.6111 | Yes |
| 9 | CTSB |  |  | 129 | 0.964 | 0.6431 | Yes |
| 10 | TNFAIP8 |  |  | 179 | 0.858 | 0.6677 | Yes |
| 11 | PRSS23 |  |  | 264 | 0.721 | 0.6761 | Yes |
| 12 | S100A11 |  |  | 275 | 0.707 | 0.7050 | Yes |
| 13 | ITGB1 |  |  | 371 | 0.615 | 0.7055 | Yes |
| 14 | LY96 |  |  | 451 | 0.547 | 0.7075 | Yes |
| 15 | FTL |  |  | 564 | 0.473 | 0.6967 | No |
| 16 | THBS1 |  |  | 613 | 0.446 | 0.7030 | No |
| 17 | VDR |  |  | 681 | 0.420 | 0.7027 | No |
| 18 | ARPC1B |  |  | 743 | 0.400 | 0.7032 | No |
| 19 | BNC2 |  |  | 864 | 0.361 | 0.6850 | No |
| 20 | CAV1 |  |  | 1341 | 0.261 | 0.5603 | No |
| 21 | GLIPR1 |  |  | 1346 | 0.260 | 0.5709 | No |
| 22 | SYNGR2 |  |  | 1939 | -0.312 | 0.4152 | No |
| 23 | CTSC |  |  | 1975 | -0.315 | 0.4194 | No |
| 24 | IL7R |  |  | 2665 | -0.460 | 0.2425 | No |
Table: GSEA details [plain text format]

  

Fig 2: WANG\_MESENCHYMAL\_PMID\_28697342: Random ES distribution      
 Gene set null distribution of ES for **WANG\_MESENCHYMAL\_PMID\_28697342**
